# Ten-Year Trends in Lithium Prescribing in Alberta, Canada

**DOI:** 10.1177/07067437231176905

**Published:** 2023-05-24

**Authors:** Samreen Shafiq, Paul Everett Ronksley, Tayler Dawn Scory, Meghan Jessica Elliott, Andrew Gabriel McKay Bulloch, Scott Burton Patten

**Affiliations:** 1 Department of Community Health Sciences, 2129 University of Calgary, Calgary, Alberta, Canada; 2 Department of Medicine, University of Calgary, Calgary, Alberta, Canada; 3 Department of Community Health Sciences, University of Calgary, Calgary, Alberta, Canada; 4 Department of Psychiatry, 2129 University of Calgary, Calgary, Alberta, Canada

**Keywords:** lithium, epidemiology, prevalence, incidence, drug utilization

## Abstract

**Aims:**

Despite lithium's clinical efficacy, it is commonly thought that its use is declining. The objective of this study is to describe the new and prevalent lithium users as well as rates of discontinuation of lithium use over a 10-year period.

**Methods:**

This study used provincial administrative health data from Alberta, Canada between January 1, 2009 and December 31, 2018. Lithium prescriptions were identified within the Pharmaceutical Information Network database. Total and subgroup specific frequencies of new and prevalent lithium use were determined over the 10-year study period. Lithium discontinuation was also estimated through survival analysis.

**Results:**

Between the calendar years of 2009 and 2018, 580,873 lithium prescriptions were dispensed in Alberta to 14,008 patients. The total number of new and prevalent lithium users appears to be decreasing over the 10-year timeframe, although the decline may have stopped or reversed in the latter years of the study period. Prevalent use of lithium was lowest among individuals between the ages of 18–24 years while the highest number of prevalent users were in the 50–64 age group, particularly among females. New lithium use was lowest amongst those 65 years and older. More than 60% (8,636) of patients prescribed lithium, discontinued use during the study timeframe. Lithium users between ages of 18–24 years were at the highest risk of discontinuations.

**Conclusions:**

Rather than a general decline in prescribing, trends in lithium use are dependent on age and sex. Further, the period soon after lithium initiation appears to be a key time period in which many lithium trials are abandoned. Detailed studies using primary data collection are needed to confirm and further explore these findings. These population-based results not only confirm a decline in lithium use, but also suggest that this may have stopped or even reversed. Population-based data on discontinuation pinpoint the period soon after initiation as the time when trials are most often discontinued.

## Abrégé

**Objectifs:**

Malgré l’efficacité clinique inégalée du lithium, on pense communément que son usage est en déclin. L’objectif de la présente étude est de décrire les nouveaux et fréquents utilisateurs du lithium ainsi que les taux de discontinuation de l’usage du lithium sur une période de dix ans.

**Méthodes:**

La présente étude a utilisé les données provinciales administratives de la santé de l’Alberta, Canada entre le 1^er^ janvier 2009 et le 31 décembre 2018. Les prescriptions de lithium ont été identifiées dans la base de données du Réseau d'information pharmaceutique. Les fréquences totales et spécifiques des sous-groupes des nouveaux utilisateurs fréquents de lithium ont été déterminées dans le cadre de l’étude de 10 ans. La discontinuation du lithium a également été estimée au moyen d’une analyse de survie.

**Résultats:**

Le nombre total d’utilisateurs nouveaux et fréquents du lithium semble décroître dans la période de 10 ans. L’usage prévalent du lithium était le plus faible chez les personnes d’entre 18 et 24 ans alors que le nombre le plus élevé d’utilisateurs prévalents était celui du groupe des 50–64 ans, en particulier chez les femmes. L’usage nouveau du lithium était le plus faible chez les personnes de 65 ans et plus. Plus de 60% des patients à qui on avait prescrit du lithium ont discontinué l’usage durant la période de l’étude. Les utilisateurs de lithium âgés de 18 à 24 ans étaient à risque élevé de discontinuations.

**Conclusions:**

Plus que du déclin général des prescriptions, les tendances de l’usage du lithium dépendent de l’âge et du sexe. En outre, la période de discontinuation du lithium semble être un moment critique pour déterminer l’accès aux bénéfices de cette médication. Ces résultats font ressortir le besoin d’interventions qui font la promotion de l’initiation et de la thérapie du lithium, particulièrement chez les jeunes patients. La période d’initiation du lithium semble être un moment critique pour déterminer l’accès aux bénéfices du lithium.

## Introduction

Lithium is currently considered first-line therapy for bipolar disorder (BD) and second-line adjunctive therapy for treatment-resistant depression.^1–3^ Prior work has shown it is especially effective for both initiation and maintenance therapy of manic and mixed episodes, with the added mitigation of suicidal behaviour.^1,4,5^ Population-level data suggests lithium use has been declining.^6,7^ This decrease in prescribing and use of lithium maybe due to reported adverse effects and therapeutic drug monitoring requirements.^8–10^ With availability of newer pharmacological treatments, physicians were given the opportunity to provide patients with other treatment options, bypassing some of lithium therapy's inconveniences and disadvantages.^11^ In addition, Malhi et al. suggests that lithium use may have decreased due to a lack of industrial and marketing support and clinical evidence derived from older studies.^12^ Because of these considerations, BD is often treated with alternate medications, which may lead to increased risk of relapse, hospitalization and suicidal ideation.^13–15^ For example, a systematic review of randomized controlled trials (RCT) by Severus et al. indicated that individuals on lithium were less likely to experience manic episode relapses compared to placebo (RR 0.52 (95% CI, 0.38 to 0.71)) and anticonvulsants (RR 0.66 (95% CI, 0.44 to 1.00)).^13^ In another study, psychiatric hospital admissions were decreased by 34% for individuals on lithium, while, head-to-head analysis indicated that lithium significantly decreased risk of hospitalizations compared to lamotrigine, quetiapine, olanzapine and carbamazepine.^14^ Lastly, a meta-review of two systematic reviews of randomized controlled trials estimated more than 60% decrease in risk of suicides and all-cause mortality for individuals on lithium compared to placebo.^15^

In Canada, a recent population-based study using data from four national surveys reported a pooled prevalence of lithium use of 0.2% (95% CI, 0.1 to 0.3%).^16^ Another study in Ontario found that only 23.4% of older adults with BD were prescribed lithium as either combination or monotherapy between April 2006 and March 2012.^17^ While these estimates suggest low use, it is not clear whether these findings suggest a change in lithium prescribing practices in Canada over time.

A decrease in lithium use has been noted in Australia between 2007 and 2015 and in Germany between 2009 and 2018.^7,18^ However, studies describing lithium prescribing in the early 2000s in Scandinavian countries and Netherlands found an increase in use.^6,19^ A recent study by Poranen et al. observed five-year medication use (from 1996 to 2022) for individuals with newly diagnosed BD.^20^ Overall, individuals were more frequently prescribed antidepressants, antipsychotics and anticonvulsants within the first three months of initial diagnosis. By the end of five-year follow-up, a decrease in use of these three medications was noted. Lithium was prescribed least frequently, however, the use remained steady within the range of 5.9–6.5%. Unfortunately, there is limited evidence to describe changes in lithium prescribing in Canada in the recent years. Further, it is unknown whether there are sex and/or age differences in the prevalence, initiation and discontinuation patterns of lithium use. Given these knowledge gaps, we aimed to describe the trends in lithium prescribing in Alberta, Canada between 2009 and 2018, with a particular interest in sex and age differences over this 10-year interval.

## Methods

### Study Design and Data Source

We obtained linked administrative health data from the Ministry of Health within Alberta, Canada between January 1, 2009 and December 31, 2018 (the latest available at the time of study initiation). This included laboratory, pharmacy and health encounter data for all adults ( ≥ 18 years). Lithium dispensation data was captured through the Pharmaceutical Information Network (PIN), which includes the majority (96%) of medications dispensed at Alberta community pharmacies but does not cover medications administered in hospitals. The PIN database includes dispensing information (i.e., date of dispensing, quantity, duration, drug form, drug identification number (DIN), and prescriber information). This data source was linked to the provincial health insurance registry, which provides information on patient sex, date of birth, migration or death status. Next, the PIN and provincial health insurance registry was linked to physician claims database. Using this database, we identified individuals with at least one International Classification of Diseases, Ninth revision (ICD-9) code for BD including 296.0, 296.1, and 296.4–8. Ethical approval was obtained from the University of Calgary's Conjoint Bioethics Review Board and granted waiver of patient consent.

### Study Population

We included all adults with a record of at least one lithium prescription within the PIN data dispensed between January 1, 2009 and December 31, 2018. Lithium prescriptions were identified through all available lithium-specific DINs (Appendix I), which are assigned to each brand and strength of medication available in Canada. All study participants were categorized into mutually exclusively groups by sex (male and female) and age group (18–24, 25–49, 50–64 and 65 years and over).

### Study Outcomes

#### Annual New and Prevalent Users

New lithium users were identified as those with first lithium prescription and no record of lithium dispensing in the prior year. We report estimates from 2010 onward to allow for a one-year look back window for all study participants. For prevalent users, we identified patients with at least one lithium prescription within a given year. This annual prevalence is reported from 2009 until 2018. The mid-year Alberta population for each year was the denominator for these calculations. For estimation of rates of new use, the prevalent users were removed from the mid-year Alberta population. Rates of new and prevalent use were reported per 100,000 persons with stratification by sex and age group.

#### Lithium Discontinuation

Lithium discontinuations were based on the last prescription fill date 1 year prior to observation end date (December 31, 2018). Therefore, individuals with last prescription fill date before January 1, 2018, were considered permanently discontinued. Total duration was calculated as the interval from the start of lithium prescribing to the last prescription fill or end of the study interval, ignoring discontinuation and restarting within this interval. The last prescription's number of days the drug was dispensed was added to this duration. This method of calculating duration accounted for differences in anticipated prescription fill dates due to changes in dosage and the actual dispensing date, which may be contingent on serum lithium levels, adverse effects or responsiveness to the treatment. Migration out of the province, death or end of the study dates were used to censor a patients’ duration of lithium therapy within the time frame of the analysis.

#### Statistical Analysis

In order to provide a smoothed description of the trends, yearly changes in total new and prevalent lithium users were initially examined using linear regression. However, linearity assumptions were found to be violated and therefore alternate modeling strategies were explored and parsimonious approaches were identified through the deviance difference test, Akaike Information Criteria and Bayesian Information Criteria for each model.^21^ In addition, each model was plotted against the scatter plot of the outcomes to visually ensure that trends were captured. In all models, fractional polynomials provided the best fit. Models were also stratified by age and sex to explore trends within subgroups over time. For each age group, plots for new and prevalent lithium users are provided, with 95% confidence intervals.

Time-to-event analysis was used to assess rates of lithium discontinuation, where failure was considered as discontinuation of lithium therapy between the time from first prescription to last prescription. Individuals were censored at the study end date if they did not discontinue lithium, out-migrated from the province or died within the 10-year period. Preliminary analysis included graphical representation of the survival function using a Kaplan-Meier (KM) plot stratified by age and sex. The proportional hazards assumption was tested using an age and sex-adjusted log-log plot (log of log survival function versus log time), goodness of fit test based on Schoenfeld's residuals and inclusion of time-dependent variables for age group and sex in the models.

## Results

### Cohort Characteristics

Between the calendar years of 2009 and 2018, 580,873 lithium prescriptions were dispensed in Alberta to 14,008 patients. Amongst these patients, demographic information was successfully linked for 13,979 patients (99.8%). During the 10-year study period, 43.5% (95% CI, 42.7 to 44.4%) of lithium-treated patients were male and 56.5% (95% CI, 55.6 to 57.3%) were female. Within this cohort, 11.9% (95% CI, 11.3 to 12.4%) were 18–24 years at the time of treatment initiation, 55.5% (95% CI, 54.7 to 56.4%) were 25–49 years, 25.3% (95% CI, 24.6 to 26.0%) were 50–64 years, and 7.3% (95% CI, 6.9 to 7.8%) were 65 and over.

There were 11,921 individuals (85.1% (95% CI, 84.5 to 85.7%)) with at least one ICD-9 code for BD. Of which 12.3% (95% CI, 11.7 to 12.9%) were from between 18 and 24 years, 56.5% (95% CI, 55.5 to 57.3%) between 25 and 49 years, 24.3% (95% CI, 23.5 to 25.0%) between 50 and 64 years, and 6.8% (95% CI, 6.4 to 7.3%) of 65 and above.

### New and Prevalent Lithium Users

**Figure 1.**
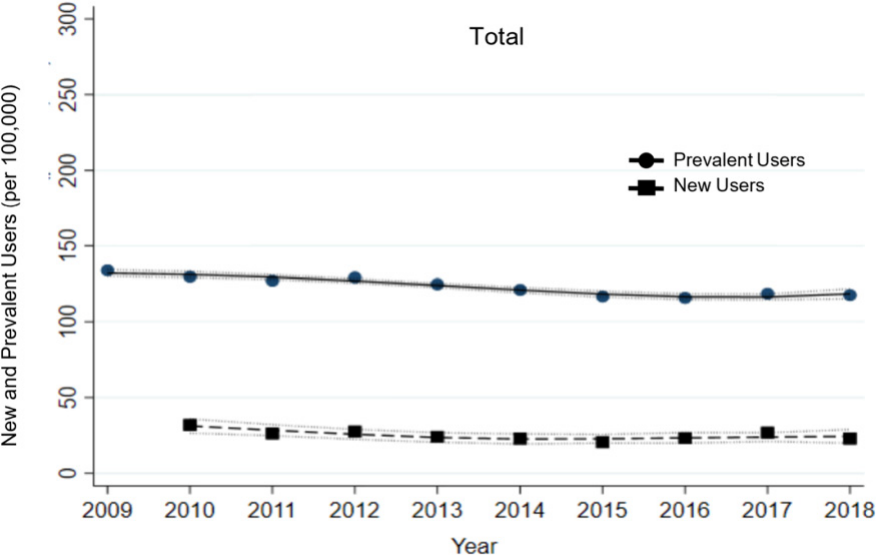
Total prevalent lithium users from 2009 to 2018 and new lithium users from 2010 to 2018.

The total population of prevalent and new users from 2009 to 2018 shows an overall decline in lithium use (Figure 1). In 2009, approximately 130 prevalent users per 100,000 are observed, while by 2017 the number of prevalent users decreased to 117 per 100,000. However, the graphic suggests that the decline had stopped or perhaps even reversed by 2018. The lack of linearity is related to a slowing or even reversal of what initially looks like a linear decline.

**Figure 2.**
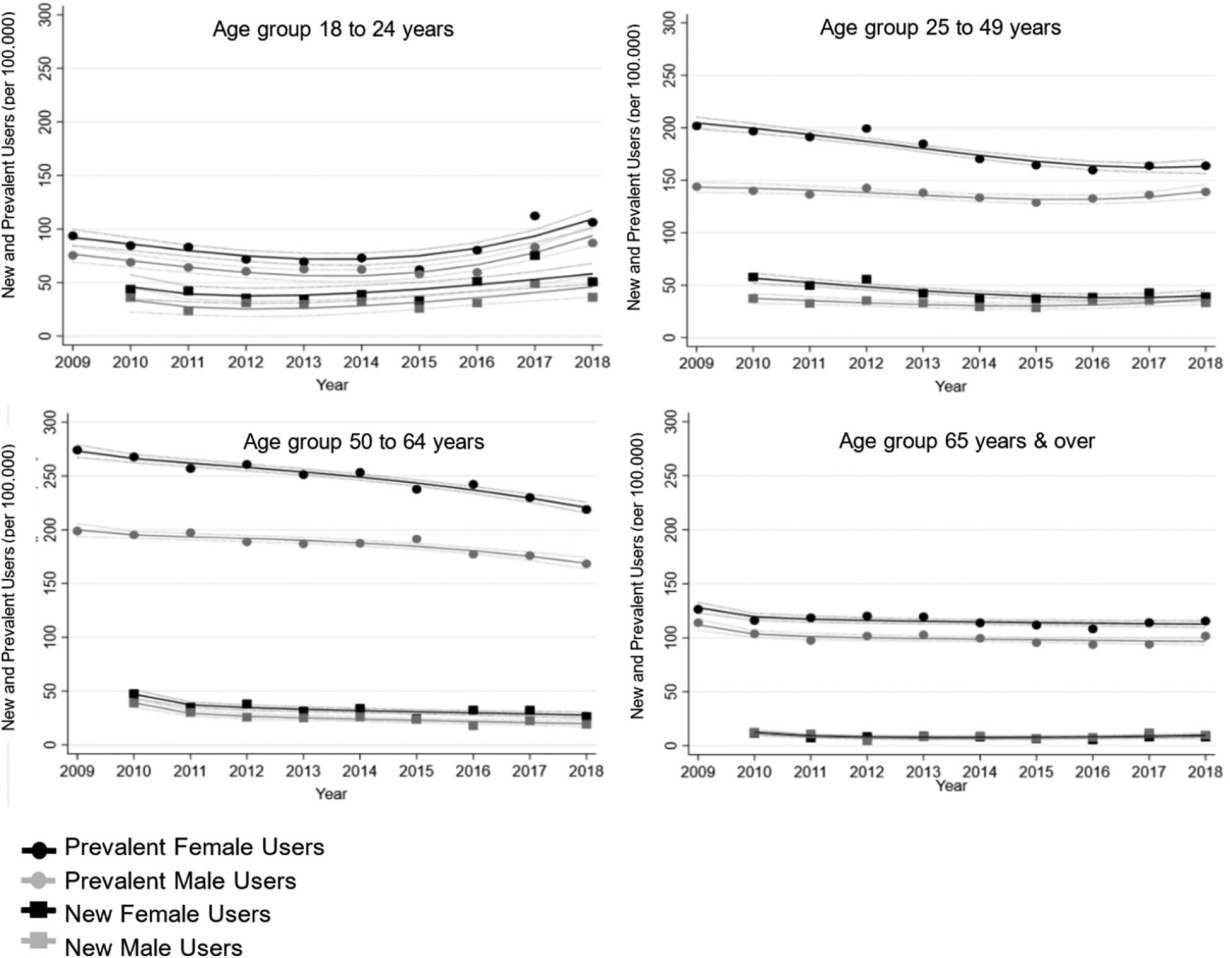
Lithium prevalent users from 2009 to 2018 and new users from 2010 to 2018 according to age groups and sex.

The new and prevalent lithium use trends stratified according to age group and sex are presented in Figure 2. Within age groups, individuals between ages 18 and 24 years had the lowest number of prevalent users over the 10-year period. The number of prevalent users remained below 100 per 100,000. However, the decrease appears to change to an increase after 2015. For patients aged 25–49 and 50–64 years, prevalent users were more often female, although, in both age groups, the numbers of female prevalent users decreased throughout the 10-year period. Amongst individuals of 65 years and above, the number of prevalent users remained stable.

The total number of annual new lithium users from 2010 to 2018 showed decreasing lithium use over time. There were almost 30 new users per 100,000 in 2010 and by 2017 the number of new users decreased to close to 20 per 100,000. The rate of new lithium use appeared similar in all age groups, except for those 65 years and older which had the lowest number of new users. Overall, the trends were similar to prevalent user trends for all age groups. Specifically, the number of new lithium users decreased and then increased amongst individuals between ages 18 and 24 years. For the 25–49 age group, the number of new users was higher in females than males. However, female new users decreased throughout the 9-year period, whereas the number of male new users appeared stable. Amongst individuals 65 years and above, the number of new lithium users appeared unchanged over the study timeframe.

### Lithium Discontinuations

**Figure 3.**
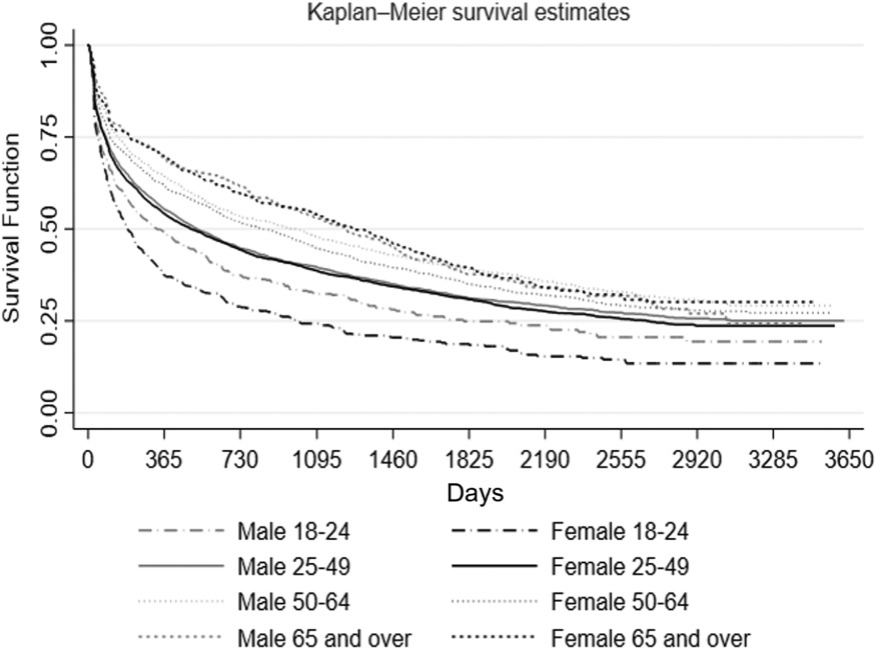
Kaplan-Meier (KM) plot for the risk of lithium discontinuation according to individual age groups.

From 2009 to 2018, 8,636 (61.6% (95% CI, 60.8 to 62.4%)) patients discontinued lithium. These individuals had at least one prescription in the 10-year time period. The discontinuation survival function is presented in the KM graph in Figure 3. The graph indicates that use declines rapidly in the first 365 days across all age groups and by sex. The steepest decline was noted for both males and females in the 18–24 year category, indicating higher discontinuation rates. In contrast, discontinuation rates were lowest for those in the oldest age group.

## Discussion

This population-based study suggests that the overall number of new and prevalent lithium users is decreasing in Alberta between the years of 2009 and 2018, but the observed pattern suggests that this decrease may have stopped by the end of our study interval. We observed five key findings: (1) Prevalent lithium use was lowest in young patients aged 18–24 years; (2) New lithium use was lowest amongst the oldest age group; (3) Prevalent use varied by sex, with higher use among females aged 25–49 and 50–64 years; and (4) More than 60% of lithium users discontinued in the 10-year period; (5) The rate of discontinuation was very high in the first year of use, especially in young people.

The total number of prevalent lithium users throughout the ten-year duration was marginally less than the previously reported Canadian pooled prevalence of 0.2% (95% CI, 0.1 to 0.3%).^16^ In this particular study, the pooled estimate of prevalent lithium use was a combination of four national surveys, while our study reports the lithium use prevalence in Alberta alone, based on pharmacy records. This may suggest that the use of lithium is slightly lower in Alberta compared to other parts of Canada. The authors of the previous study also noted a chronological decline in the prevalence estimates from the four national surveys.^16^ This finding is corroborated in our study, where the prevalence of use decreased from 2009 to 2018, although the trend was not linear. In addition, other studies investigating similar time periods indicate a decrease in lithium use prevalence, as noted above.^7,18^ In our study, the total number of new lithium users remained below 50 per 100,000 persons during the ten-year period. Stratification by age and sex suggest a decrease in new lithium use comparable to prevalent lithium use. This finding suggests that these changes are predominantly driven by the decrease in new use of lithium, whereas the mean duration of lithium has remained more constant (note that the prevalence of use is approximately the product of the rate of new use and the average duration of use). Moreover, discontinuations occur frequently amongst lithium users, especially near the time of treatment initiation. Discontinuation, in the absence of new use of lithium affects the prevalence steady-state.

The lowest prevalence of lithium use was observed in the youngest age group (18–24 years). Similar findings were noted in a study conducted in Norway, Sweden and Denmark in 2006.^6^ In these countries, the prevalence was less than 1 per 1000 population amongst individuals aged 18–24 years. Additionally, lithium discontinuation was highest in this age group, particularly in females. Lithium discontinuation may occur due to adverse effects, the lack of beneficial effects or lack of compliance or understanding of lithium therapy and BD.^10^ Even so, this age group is especially vulnerable to poor treatment and disease outcomes.^3^ Lower lithium initiation in the youngest age group maybe due to delayed recognition of BD, as initial presentation is often of major depressive disorder or hypomanic symptoms.^22^

On the other hand, our study indicates that the trend of new and prevalent lithium users follows a U-shaped pattern in the youngest age group, suggesting that although lithium use was decreasing until 2014, its use may be increasing in more recent years. This may be due to better access to specialized care or monitoring, or, for other reasons such as personal preferences, failed trials of other medications or tolerability issues with alternative mood stabilizers. Even though, an increasing trend of lithium use is noted, discontinuation rates are high amongst the youngest group. Further studies are needed to understand the factors that impact lithium discontinuations and compliance. Given the limitations of administrative data, such studies may best make use of primary data collection and/or qualitative methods. Identification of factors that are associated with lithium discontinuations may help to identify groups for whom some lithium discontinuations may be unnecessary or undesirable.

In our study, the number of prevalent users steadily declined in the 50 to 64 age group, while the number of new lithium users was also low and declining. This may indicate discontinuation of lithium due to adverse effects (such as renal or thyroid dysfunction), preferences for other medications or non-response. Again, more detailed studies using other data sources will be needed to address these questions.

Prior work has similarly found that lithium incidence was lowest in the youngest and then the oldest age groups.^6^ Lithium is recommended as first-line therapy in elderly patients with BDs, with additional monitoring recommended by guidelines, which also mention possible benefits for cognitive function in older patients.^3^ An encouraging observation from our study is that older individuals are least likely to discontinue lithium, despite this being the age where renal issues are most likely to emerge. Notably, much of the literature is concerned with discontinuation later in life due to long-term adverse effects, whereas the steep discontinuation curve early in life suggests possible opportunities to optimize lithium prescribing by identifying opportunities (through e.g., adjunctive psycho-education, support with side-effect management) soon after initiation of lithium therapy.

There were several limitations in this study. First, we have provided a number of individuals with ICD-9 code for BD within the lithium users cohort. This number should be interpreted with caution due to a lack of validated case definition for BD for administrative data.^23,24^ In addition, we used some ICD-9 codes that may also have included major depressive disorder in this study (“296” ICD-9 codes). This limitation is difficult to overcome, as BD is a complex condition and diagnosis depends on subjective recordings of symptoms, which often delays diagnosis.^25^ Second, although the PIN database captures 96% of prescription dispensed in community pharmacies, it does not confirm that prescriptions dispensed were used by the patients. This may result in overestimation of the number of new and prevalent lithium users. Third, our definition of discontinuation was based on the last prescription in the patients’ record. Multiple discontinuations may have occurred during the course of lithium therapy, which may have distorted the total duration of treatment in our study. Like most studies based on administrative data, our goal was to estimate broad trends within the population, rather than the precise dynamic of lithium therapy in individual patients. Even so, our study focuses on permanent discontinuations, which occurred in more than half of the study population. In addition, we were not able to capture whether lithium was used in combination or monotherapy. We aim to observe these trends with more recent use in future studies. Lastly, we were not able to capture lithium initiation that occurred in hospitals. Initial acute mania is often diagnosed and managed in hospital in most cases and the first post-discharge outpatient prescription would have been captured by the PIN database. This may lead to erroneous yearly incidence estimates, where the number of new lithium users were captured much later than actual initial use.

In conclusion, trends in lithium use are dependent on age and sex. The period soon after lithium initiation appears to be a critical time for determining continuation of treatment. Detailed studies using primary data collection are needed to confirm and further explore these findings. These population-based results not only confirm a decline in lithium use, but also suggest that this may have stopped or even reversed. Population-based data on discontinuation pinpoint the period soon after initiation as the time when trials are most often discontinued. Further studies are also required to better understand factors that are associated with lithium discontinuation and non-compliance.

## Supplemental Material

sj-docx-1-cpa-10.1177_07067437231176905 - Supplemental material for The Canadian Journal of Psychiatry Click here for additional data file. Supplemental material, sj-docx-1-cpa-10.1177_07067437231176905 for The Canadian Journal of Psychiatry by Samreen Shafiq, BPharm, MSc, Paul Everett Ronksley, PhD, Tayler Dawn Scory, MSc, Meghan Jessica Elliott, MD, MSc, Andrew Gabriel McKay Bulloch, PhD and Scott Burton Patten, MD, PhD in The Canadian Journal of Psychiatry
