# Supplementary material for: Ten-Year Trends in Lithium Prescribing in Alberta, Canada
Source: Can J Psychiatry. 2023 May 24;69(1):13–20. doi: 10.1177/07067437231176905 (PMC10867410; doi:10.1177/07067437231176905)
Supplement: sj-docx-1-cpa-10.1177_07067437231176905 - Supplemental material for The Canadian Journal of Psychiatry [file sj-docx-1-cpa-10.1177_07067437231176905.docx]

# Supplementary Material

## Appendix I

| **Table 1: Lithium Drug Identification Number (DIN) available in Canada** | | | |
| --- | --- | --- | --- |
| **Status** | **DIN** | **Product Name** | **Active Ingredient and strength** |
| Marketed | 02242837 | Apo-Lithium Carbonate | Lithium Carbonate 150 Mg |
| Marketed | 02242838 | Apo-Lithium Carbonate | Lithium Carbonate 300 Mg |
| Marketed | 00236683 | Carbolith | Lithium Carbonate 300 Mg |
| Marketed | 00461733 | Carbolith | Lithium Carbonate 150 Mg |
| Marketed | 02011239 | Carbolith | Lithium Carbonate 600 Mg |
| Approved | 02231397 | Dom-Lithium Carbonate | Lithium Carbonate 150 Mg / Cap |
| Approved | 02231398 | Dom-Lithium Carbonate | Lithium Carbonate 300 Mg / Cap |
| Approved | 02231399 | Dom-Lithium Carbonate | Lithium Carbonate 600 Mg / Cap |
| Cancelled Post Market | 00590665 | Duralith Tab 300mg | Lithium Carbonate 300 Mg |
| Cancelled Post Market | 02304511 | Euro Lithium | Lithium Carbonate 150 Mg |
| Cancelled Post Market | 02304538 | Euro Lithium | Lithium Carbonate 300 Mg |
| Marketed | 00406775 | Lithane | Lithium Carbonate 300 Mg |
| Marketed | 02013231 | Lithane | Lithium Carbonate 150 Mg |
| Cancelled Post Market | 00328782 | Lithizine Cap 150mg | Lithium Carbonate 150 Mg / Cap |
| Cancelled Post Market | 00328790 | Lithizine Cap 300mg | Lithium Carbonate 300 Mg / Cap |
| Marketed | 02266695 | Lithmax | Lithium Carbonate 300 Mg |
| Cancelled Post Market | 02237441 | Pal-Lithium Carbonate | Lithium Carbonate 150 Mg |
| Cancelled Post Market | 02237442 | Pal-Lithium Carbonate | Lithium Carbonate 300 Mg / Cap |
| Cancelled Post Market | 02237443 | Pal-Lithium Carbonate | Lithium Carbonate 600 Mg / Cap |
| Cancelled Post Market | 02237006 | Phl-Lithium Carbonate | Lithium Carbonate 150 Mg |
| Cancelled Post Market | 02237007 | Phl-Lithium Carbonate | Lithium Carbonate 300 Mg |
| Cancelled Post Market | 02237008 | Phl-Lithium Carbonate | Lithium Carbonate 600 Mg |
| Marketed | 02216132 | Pms-Lithium Carbonate - Cap 150mg | Lithium Carbonate 150 Mg |
| Marketed | 02216140 | Pms-Lithium Carbonate - Cap 300mg | Lithium Carbonate 300 Mg |
| Marketed | 02216159 | Pms-Lithium Carbonate - Cap 600mg | Lithium Carbonate 600 Mg |
| Dormant | 02074834 | Pms-Lithium Citrate | Lithium (Lithium Citrate) 8 Mmol / 5 Ml |
